# Phospholipid metabolism regulates AML growth and stemness

**DOI:** 10.18632/aging.102055

**Published:** 2019-06-25

**Authors:** Mingjing Xu, Ayesh K. Seneviratne, Aaron D. Schimmer

**Affiliations:** 1 Princess Margaret Cancer Centre, University Health Network, Toronto, ON, Canada

**Keywords:** acute myeloid leukemia, leukemia stem cell, mitochondria, phospholipid metabolism, phosphatidylserine

Emerging evidence has shown that mitochondrial pathways are potential therapeutic targets for cancers. Mitochondria are the central organelles for energy production, but also play a pivotal role in lipid and amino acid metabolism, redox and calcium homeostasis, transcription regulation, as well as cell fate determination. Previously we and others have demonstrated that disruption of mitochondrial function selectively targets acute myeloid leukemia (AML) cells [1,2]. Identification of key mitochondrial components of AML cells may thus elicit more effective therapies.

In our recent publication in Cell Stem Cell [3], we utilized a genome-wide Clustered Regularly Interspaced Palindromic Repeats (CRISPR) screen to identify mitochondrial genes that are essential for AML viability and growth. In this screen, we identified tafazzin (TAZ) that ranked in the top 1% of mitochondrial hits. TAZ is a mitochondrial acyltransferase that catalyzes the maturation of cardiolipin, the main phospholipid in the inner mitochondrial membrane. Congenital TAZ mutations are associated with Barth’s syndrome [4], but roles of TAZ in AML and other cancers remain largely unknown.

We interrogated the function of TAZ in AML through a series of functional assays. Loss of TAZ reduced proliferation, supressed clonogenic growth and induced differentiation in AML cells. Of note, knocking down TAZ in mice did not affect normal hematopoiesis, suggesting a specific dependence of AML cells on TAZ.

As TAZ is a key enzyme for phospholipid biosynthesis, we examined the phospholipid profile of AML cells upon TAZ knockdown. In addition to the expected decrease in mature cardiolipin, we noticed increased levels of phosphatidylserine (PS) and decreased phosphatidylethanolamine (PE). Further study showed that supplementing TAZ knockdown cells with PE did not rescue the loss in proliferation. In contrast, supplementation of PS mimicked the effects of TAZ knockdown and decreased AML growth and stemness.

Furthermore, phosphatidylserine decarboxylase (PISD), a mitochondrial enzyme that converts PS to PE, was downregulated at post-transcriptional level following TAZ knockdown. By lipid-protein overlay assay, we found that PISD recombinant protein bound cardiolipin and the cardiolipin moiety phosphatidylglycerol. CRISPR/Cas9-mediated knockout and chemical inhibition of PISD recapitulated the effects of knockdown of TAZ. Taken together, we discovered that TAZ modulates PISD activity to control intracellular levels of PS, which in turn regulates AML stemness and differentiation.

Mechanically, we discovered that TAZ knockdown or increasing levels of PS up-regulated genes in the Toll-Like Receptor (TLR) pathway. We also showed that activation of the TLR pathways was functionally important to induce AML differentiation. Finally we used preclinical animal models to demonstrate that PISD inhibitor, MMV007285, reduced AML disease burden and targeted AML stem cells without toxicity.

This work provides new insights into the roles of TAZ in AML, and highlights the important role of PS in maintaining AML stemness. PS is an essential phospholipid which is normally restricted to the inner leaflet of the cell membrane. Previous studies on PS focused on the translocation of PS to the cell surface. For example, the externalization of PS has been considered as a hallmark of apoptosis [5]. Exposed PS is an immunosuppressive signal in infectious disease and cancer [5]. However, little is known about the function of intracellular PS. Our data showed that TAZ knockdown increased intracellular PS, but did not alter the PS level on cell surface. By supplementing cell medium with PS, we increased intracellular PS level and observed reduced colonogenic growth *in vitro* and less leukemic engraftment *in vivo*. We also discovered that intracellular PS inhibited AML growth and stemness by activating the TLR pathway. This finding implicates a novel function of PS in regulating cell growth and stemness. It also drives further studies on how PS mediates the TLR activation and the roles of inflammatory pathways in AML differentiation.

At the therapeutic level, this paper proposes a novel mechanism to target leukemia stem cells (LSCs), which are the subpopulation of cells responsible for AML progression and relapse. Previous studies have shown that LSCs display unique metabolic properties, including relatively low levels of reactive oxygen species [6], sensitivity to disruption of electron transport chain [2] and dependence on amino acid metabolism [7]. Thus, targeting these metabolic mechanisms would likely result in selective toxicity to LSCs. Our data showed that increasing levels of PS impeded the clonogenic growth of primary AML cells over normal hematopoietic cells. In animal models, a PISD inhibitor targeted LSCs as evidenced by the reduced engraftment of primary AML cells in secondary transplant. Intriguingly, a recent study from Keckesova et al. [8] also showed that mitochondrial phospholipid biosynthesis is required for the growth and stemness of breast cancer cells. Both studies implicated phospholipid metabolism as a new metabolic vulnerability of cancer cells.

**Figure 1:**
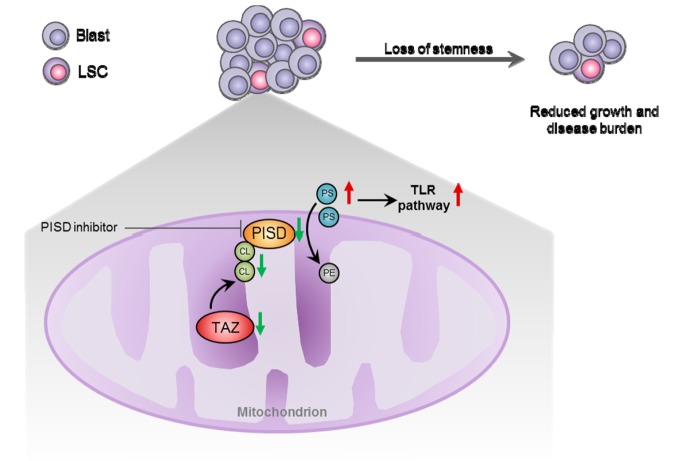
**Phospholipid metabolism regulates AML growth and stemness.** Loss of tafazzin (TAZ) reduces cardiolipin (CL) level and causes subsequent dysfunction of phosphatidylserine decarboxylase (PISD), which in turn increases intracellular levels of PS. PS suppresses acute myeloid leukemia (AML) stemness and induces AML differentiation through activation of Toll-Like Receptor (TLR) pathway. PISD inhibitor, genetic knockout of PISD or supplementation of PS result in the same therapeutic effect, suggesting increasing PS is a potential therapeutic strategy for AML.

In summary (Figure 1), we discovered that TAZ is critical for AML growth and stemness. By studying the underlying mechanisms, we uncovered a novel link between phospholipid metabolism and AML stemness, thereby providing a potential therapeutic target for AML. Further investigation on phospholipid metabolism will be important to develop a more comprehensive picture of metabolic properties of AML and identify new therapeutic strategies.
